# Nanoparticle albumin‐bound paclitaxel in elder patients with advanced squamous non‐small‐cell lung cancer: A retrospective study

**DOI:** 10.1002/cam4.2791

**Published:** 2019-12-26

**Authors:** Yang Liu, Yinping Dong, Hui Zhu, Wang Jing, Hongbo Guo, Jinming Yu

**Affiliations:** ^1^ Lung Cancer Center West China Hospital Sichuan University Chengdu China; ^2^ Department of Radiation Oncology Shandong Cancer Hospital and Institute Shandong First Medical University and Shandong Academy of Medical Sciences Jinan China; ^3^ Department of Gastric Cancer Tianjin Medical University Cancer Institute & Hospital Tianjin China; ^4^ Department of Thoracic Surgery Shandong Cancer Hospital and Institute Shandong First Medical University and Shandong Academy of Medical Sciences Jinan China

**Keywords:** adverse events, elderly, nab‐PTX, squamous NSCLC, survival

## Abstract

**Purpose:**

This study aimed to assess the effect of nanoparticle albumin‐bound paclitaxel (nab‐PTX) chemotherapy regimens in elderly patients (≥70 years old) with advanced squamous non‐small‐cell lung cancer (NSCLC).

**Patients and Methods:**

The clinical records of elderly patients aged ≥70 years with advanced squamous NSCLC were reviewed retrospectively. All of these patients received nab‐PTX, with or without combination of chemotherapy in Shandong Cancer Hospital and Institute between 1 July 2012 and 30 June 2017. We analyzed the toxicity profiles, progression‐free survival (PFS), overall survival (OS), objective response rate (ORR), and disease control rate (DCR).

**Results:**

Totally, 52 elderly patients with squamous NSCLC were included in the analysis. For all patients, the ORR was 34.6%, the DCR was 80.8%, median PFS was 5.9 months (95% confidence interval [CI]: 4.0‐7.8 months), and median OS was 14.3 months (95% CI: 11.0‐17.8 months). Combination with chemotherapy significantly prolonged OS (19.3 vs 11.2 months, *P* = .016), despite a nonsignificant improvement in PFS (7.1 vs 4.2 months, *P* = .060) vs monotherapy. For patients who received nab‐PTX as first‐line treatment, the median PFS and OS were 6.7 months and 17.2 months, respectively, and the median OS in combination therapy subgroup was significantly higher than that in monotherapy group (20.3 vs 11.2 months, *P* = .013). Meanwhile, the median PFS and OS of patients with nab‐PTX as second‐ or later‐line treatment were 4.4 months and 13.3 months, respectively, but no survival benefit was achieved by the combination chemotherapy when compared with single‐agent chemotherapy. Hematologic toxicities were the most common adverse events (AEs), which include grade 3 or 4 neutropenia (13.7%), thrombocytopenia (4.1%), and anemia (6.8%). The main nonhematologic toxicities were peripheral sensory neuropathy (39.7%), followed by anorexia and nausea/vomiting.

**Conclusion:**

In elderly advanced squamous NSCLC patients, the treatment of nab‐PTX was effective and well tolerated.

## Graphical abstract

Elderly patients account for the majority of all lung cancer cases, and these patients have a greater mortality rate. In this study, advanced squamous non‐small‐cell lung cancer patients aged ≥70 years who received nab‐PTX, with or without combination of chemotherapy, have been retrospectively analyzed. The use of nab‐PTX, either as first‐line or later‐line chemotherapy, was found to be effective and well tolerated.

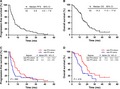

## 1 INTRODUCTION

Non‐small‐cell lung cancer (NSCLC) is the most common lung cancer subtype, and squamous cell carcinoma (SCC) accounts for 30% of NSCLC.^1^, ^2^, ^3^ The prognosis of advanced SCC, when compared with lung adenocarcinoma, is poor, with the 5‐year survival rate <5%.^4^, ^5^ Although significant progress in the treatment of NSCLC has been made in recent years, including target treatment with inhibitors for epidermal growth factor receptor (EGFR) and ALK tyrosine‐kinase, due to low frequency of EGFR mutations and ALK rearrangements, the treatments were less effective in squamous NSCLC.^6^, ^7^ Therefore, the preferred first‐line treatments for unresectable advanced SCC patients are still platinum‐based chemotherapy.^8^

Elderly patients account for the majority of all lung cancer cases,^9^ and these patients have a greater mortality rate.^10^ In consideration of comorbidities, poorer general health status, and anticipated intolerance to the toxicities of platinum‐based chemotherapy, oncologists often remain cautious about treatment regimens in elderly patients, especially those ≥70 years old, leading to undertreatment in these population.^11^, ^12^ Thus, for elderly patients with lung SCC, more effective and tolerable treatment options are in need; however, few studies have been conducted to explore suitable treatments.

Nanoparticle albumin‐bound paclitaxel (nab‐PTX) is a novel nanoparticulate formulation of paclitaxel binding to human serum albumin. Nab‐PTX reaches tumor microenvironment more efficiently and has higher binding affinities to cancer cells when compared with solvent‐based paclitaxel (sb‐PTX). Preclinical studies have shown that binding to albumin resulted in a 10‐fold higher mean free paclitaxel concentration in serum, and a 33% greater drug concentration in xenograft tumors than sb‐PTX, suggesting higher tumor‐killing potential.^13^, ^14^ In addition, there would be less allergic reactions caused by polyoxyethylene castor oil/ethanol, the cosolvent of sb‐PTX. Numerous studies have shown the nab‐PTX's efficacy and safety in treating pancreatic cancer, ovarian cancer, breast cancer, and NSCLC.^15^, ^16^, ^17^, ^18^ In a phase III trial, which was performed by Socinski et al, nab‐PTX/carboplatin (nab‐P/C) and sb‐PTX/carboplatin (sb‐P/C) were compared as advanced NSCLC's first‐line treatment regimens. The treatment group of nab‐P/C achieved a higher objective response rate (ORR; 33% vs 25%; *P* = .005) and resulted in less neuropathy than sb‐P/C group.^19^ The results of subgroup analysis in squamous NSCLC patients also showed that the ORR in the arm of nab‐P/C was higher (41% vs 24%, *P* < .001), while the increase of overall survival (OS) was not significant (10.7 vs 9.5 months, *P* = .284).^20^ Furthermore, subgroup analysis in ≥70 years old patients identified significant improvement of median OS with nab‐P/C (19.9 vs 10.4 months, *P* = .009), and the adverse events (AEs) of nab‐P/C were better tolerated.^21^

On the basis of these clinical data, we hypothesized that nab‐PTX might be effective and well tolerated by elderly advanced squamous NSCLC patients with ages ≥70 years. Studies, including both trials and real‐world evidence on the treatment of nab‐PTX for elder squamous NSCLC patients, are currently limited. In this study, we retrospectively analyzed the clinical information of patients aged ≥70 years with advanced squamous NSCLC who had received the treatment of nab‐PTX in our hospital from 1 July 2012 to 30 June 2017, aiming to evaluate nab‐PTX's effectiveness in elderly squamous NSCLC patients as first‐ or later‐line treatment.

## 2 PATIENTS AND METHODS

### 2.1 Patient selection

We retrospectively reviewed clinical records of elderly patients aged ≥70 years with advanced lung SCC who received nab‐PTX in chemotherapy regimens between 1 July 2012 to 30 June 2017 in Shandong Cancer Hospital and Institute. The inclusion criteria were as follows: histologically confirmed squamous NSCLC; ages ≥70; performance status of 0 to 2 for Eastern Cooperative Oncology Group (ECOG); lesions that can be measured by the Response Evaluation Criteria in Solid Tumors (RECIST) version 1.1; received either nab‐PTX monotherapy or combined with other chemotherapy agents. Patients were excluded if they had serious complications, poorly controlled symptomatic brain metastases, or severe heart disease.

### 2.2 Data collection

Baseline demographic and clinical information were extracted from clinical records. Baseline characteristics collected included basic demographic characteristics, smoking, and drinking history. Disease was staged for all patients at the diagnosis based on the TNM classification for lung cancer with eighth edition.^22^ Other clinical data including histological type, laboratory tests, and imaging data were also obtained. Outcome data were either extracted from available information records or further acquired with telephone follow‐up. The last time of follow‐up was 10 May 2018. This study was approved by the institutional Review Board of Shandong Cancer Hospital and Institute and the informed consents were deemed waivable.

### 2.3 Efficacy and safety outcomes

The efficacy was evaluated by computed tomography (CT) every two cycles during chemotherapy. Treatment response, including complete remission (CR), partial response (PR), stable disease (SD), and disease progression (PD), was assessed based on the RECIST 1.1.^23^ ORR was calculated as the percentage of patients ever achieved CR/PR. The percentage of CR/PR/SD patients were used to calculate disease control rate (DCR). The time from the first administration of the nab‐PTX to the date of confirmation of PD or death was defined as progression‐free survival (PFS). And, the time from the first administration of the nab‐PTX‐based chemotherapy to the date of death was defined as OS. The Common Terminology Criteria for Adverse Events (CTCAE) version 4.0 was used to record and grade the AEs.

### 2.4 Statistical analyses

Kaplan‐Meier method was used to estimate the median probabilities and 95% confidence intervals (CIs) of PFS and OS. The difference in survival was compared by Log‐rank tests. *P < *.05 was defined as the statistically significant criterion, and all statistical tests were two‐sided. All analyses were performed using SPSS software (version 17.0).

## 3 RESULTS

### 3.1 Patients' characteristics and clinical information

Totally, 52 patients (49 men and 3 women) were included, with a median age was 73 (range 70‐84). Table 1 showed patients’ baseline characteristics. All patients exhibited a 0 to 2 ECOG performance status including 41 smokers (78.8%) and 28 drinkers (53.8%). Most patients were in advanced stages with 5 (9.6%) IIIA; 12 (23.1%) IIIB; 4 (7.7%) IIIC, and 31 (59.6%) stage IV. There were eight (15.1%) patients with lung metastases, six (11.3%) with brain metastases, five (9.4%) with liver metastases, 12 (22.6%) with bone metastases, and 10 (18.9%) with metastases in other organs.

**Table 1:** Patient characteristics

Baseline characteristics	No. of patients (n = 52)	%
Age, y		
Median	73	
Range	70‐84	
Sex		
Male	49	94.2
Female	3	5.8
ECOG performance status		
1	49	94.2
2	3	5.8
Smoking status		
Ever	41	78.8
Never	11	21.2
Drinking status		
Ever	28	53.8
Never	24	46.2
Clinic stage		
IIIA	5	9.6
IIIB	12	23.1
IIIC	4	7.7
IV	31	59.6
Metastatic lesion sites		
Lung	8	15.1
Brain	6	11.3
Liver	5	9.4
Bone	12	22.6
Other	10	18.9
Prior line of chemotherapy		
0	28	53.8
1	17	32.7
2	3	5.8
3	4	7.7

Abbreviations: ECOG, eastern cooperative oncology group.

Table 2 summarized the details of all nab‐PTX‐based chemotherapy regimens. All patients received nab‐PTX‐based chemotherapy at least two cycles until the progression of disease, unacceptable toxicity occurrence, or patients' retreat. The decision of regimen selection, dose adjustment, suspension, and resume were made by the physician in charge based on the patient condition. Among these 52 patients, 22 (42.3%) received monotherapy with nab‐PTX and 30 (57.7%) received nab‐PTX‐based combination chemotherapy regimens. In combination therapies, 13 (25.0%) were cotreated with cisplatin, nine (17.3%) with carboplatin, six (11.5%) with nedaplatin, one (1.9%) with gemcitabine, and one (1.9%) with vinorelbine.

**Table 2:** Treatment schedule for the patients

Treatment schedule	No. of patients	%
*nab*‐PTX monotherapy		
*nab*‐PTX 260 mg/m^2^, D1; q3 wk	4	7.7
*nab*‐PTX 100 mg/m^2^, D1,8; q3 wk	7	13.5
*nab*‐PTX 100 mg/m^2^, D1,8,15; q4 wk	11	21.2
*nab*‐PTX combined therapy		
*nab*‐PTX 100 mg/m^2^, D1,8 + DDP 75 mg/m^2^,d1; q3wk	13	25.0
*nab*‐PTX 100 mg/m^2^, D1,8 + CBP AUC:6,d1; q3 wk	9	17.3
*nab*‐PTX 100 mg/m^2^, D1,8 + NDP 100 mg/m^2^,d1; q3 wk	6	11.5
*nab*‐PTX 260 mg/m^2^, D1 + GEM 1400 mg/m^2^,d1,8; q3 wk	1	1.9
*nab*‐PTX 260 mg/m^2^, D1 + NVB 30 mg/m^2^,d1,8; q3 wk	1	1.9

Abbreviations: CBP, carboplatin; DDP, cisplatin; GEM, gemcitabine; *nab*‐PTX, nanoparticle albumin‐bound paclitaxel; NDP, nedaplatin; NVB, vinorelbine.

### 3.2 Efficacy outcomes

As shown in Table 3, during treatment in the study period, no patient achieved CR, 18 (34.6%) patients had PR, 24 (46.2%) patients were with SD, and 10 (19.2%) patients had PD. The ORR was 34.6% and the DCR was 80.8%. In subgroup analysis, the ORR and DCR were 31.8% and 72.7% (0 CR, 7 PR, 9 SD, 6 PD) for patients with monotherapy and 36.7% and 86.7% (0 CR, 11 PR, 15 SD, 4 PD) for patients with combination chemotherapy. In addition, for patients who received nab‐PTX as first‐line treatment, the ORR and DCR were 35.7% and 85.7% (0 CR, 10 PR, 14 SD, 4 PD), and for patients with nab‐PTX as second‐ or later‐line treatment, the ORR and DCR were 33.3% and 75.0% (0 CR, 8 PR, 10 SD, 6 PD), respectively.

**Table 3:** Response to nanoparticle albumin‐bound paclitaxel

Type of response	nab‐PTX alone (n = 22)		nab‐PTX + others (n = 30)		nab‐PTX as first‐line treatment (n = 28)		nab‐PTX as second‐line or later treatment (n = 24)		Total (n = 52)	
	No.	%	No.	%	No.	%	No.	%	No.	%
CR	0		0		0		0		0	
PR	7	31.8	11	36.7	10	35.7	8	33.3	18	34.6
SD	9	40.9	15	50.0	14	50.0	10	41.7	24	46.2
PD	6	27.3	4	13.3	4	14.3	6	25.0	10	19.2
ORR	7	31.8	11	36.7	10	35.7	8	33.3	18	34.6
DCR	16	72.7	26	86.7	24	85.7	18	75.0	42	80.8

Abbreviations: CR, complete remission; DCR, disease control rate; ORR, objective response rate; PD, disease progression; PR, partial response; SD, stable disease.

**Figure 1:**
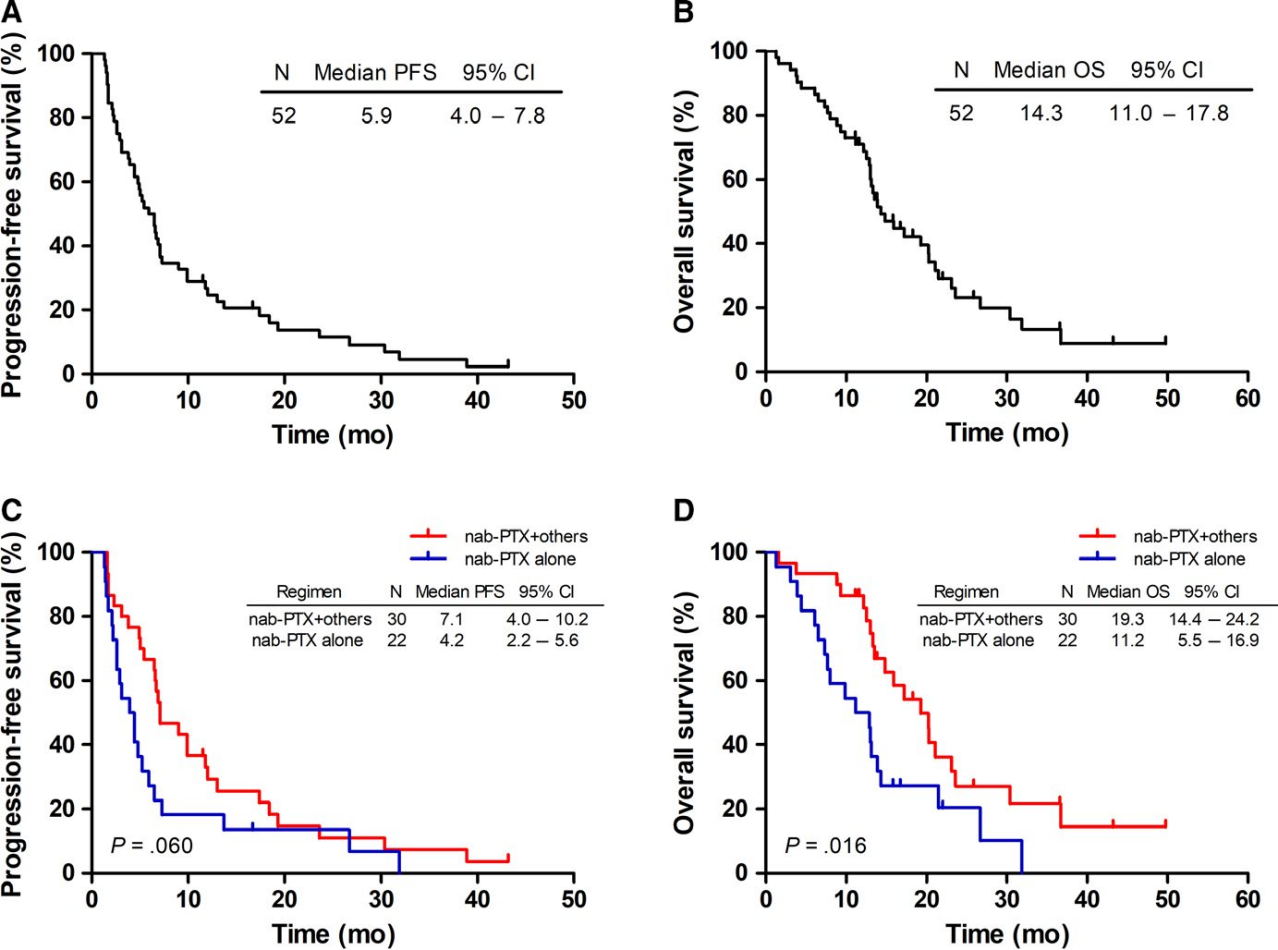
Kaplan‐Meier curves of progression‐free survival (PFS) and overall survival (OS) for all patients. A, The PFS curves for all patients. B, The OS curves for all patients. C, The PFS curves for patients who received nab‐PTX monotherapy or combination chemotherapy. D, The OS curves for patients who received nab‐PTX monotherapy or combination chemotherapy

Survival of the patients are depicted in Figure 1. The median PFS and OS of studied patients were 5.9 months (95% CI: 4.0‐7.8 months) and 14.3 months (95% CI: 11.0‐17.8 months), respectively. In subgroup analysis, the median PFS of patients who underwent monotherapy was 4.2 months (95% CI: 2.2‐5.6 months), vs 7.1 months (95% CI: 4.0‐10.2 months) in patients with combination treatment (*P* = .060). The median OS was 11.2 months (95% CI: 5.5‐16.9 months) in patients with monotherapy, compared to 19.3 months (95% CI: 14.4‐24.2 months) in patients with combination chemotherapy (*P* = .016).

**Figure 2:**
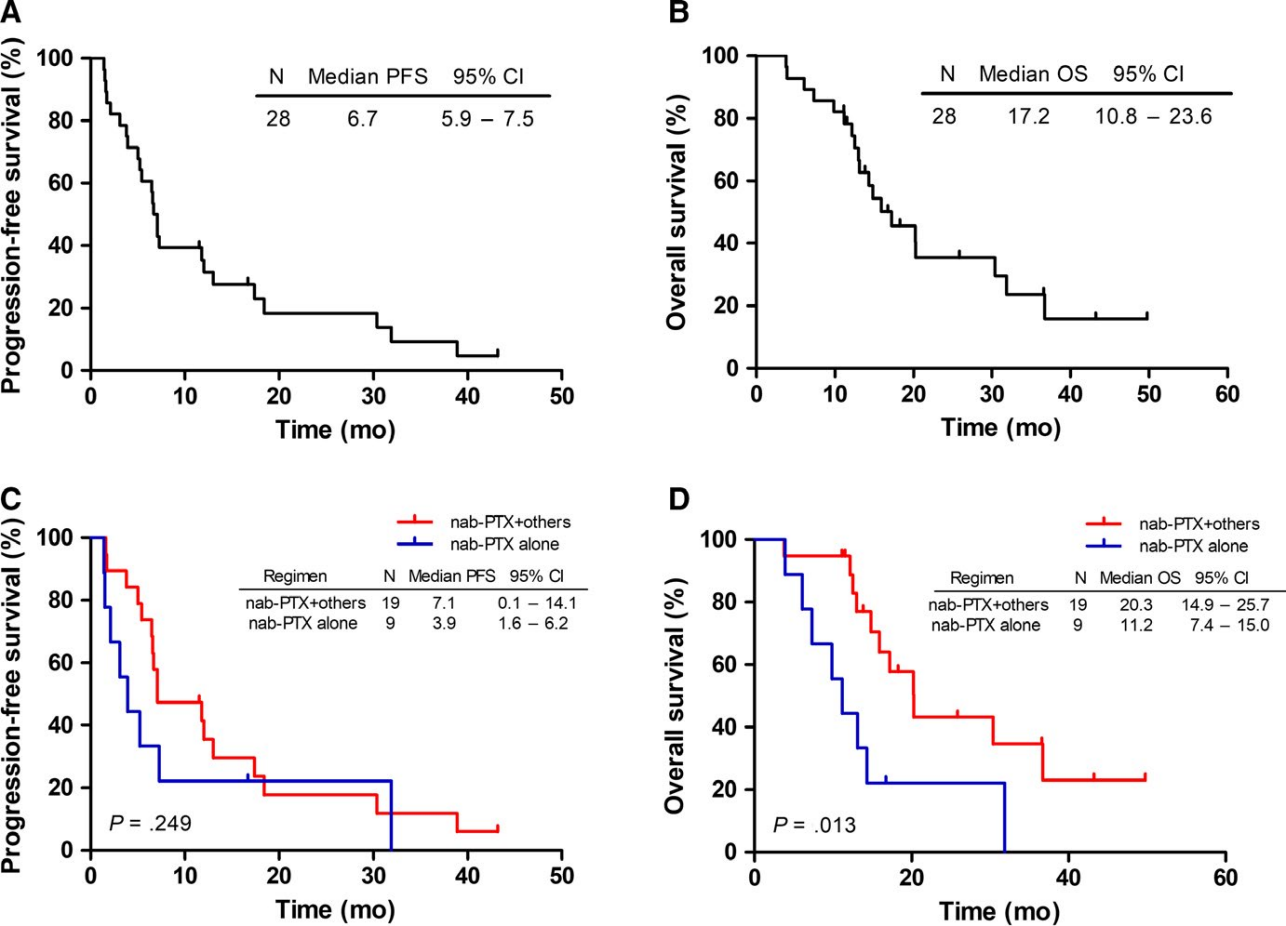
Kaplan‐Meier curves of progression‐free survival (PFS) and overall survival (OS) for patients who received nab‐PTX as first‐line treatment. A, The PFS curves for these subgroup patients. B, The OS curves for these subgroup patients. C, The PFS curves for patients who received nab‐PTX monotherapy or combination chemotherapy. D, The OS curves for patients who received nab‐PTX monotherapy or combination chemotherapy

**Figure 3:**
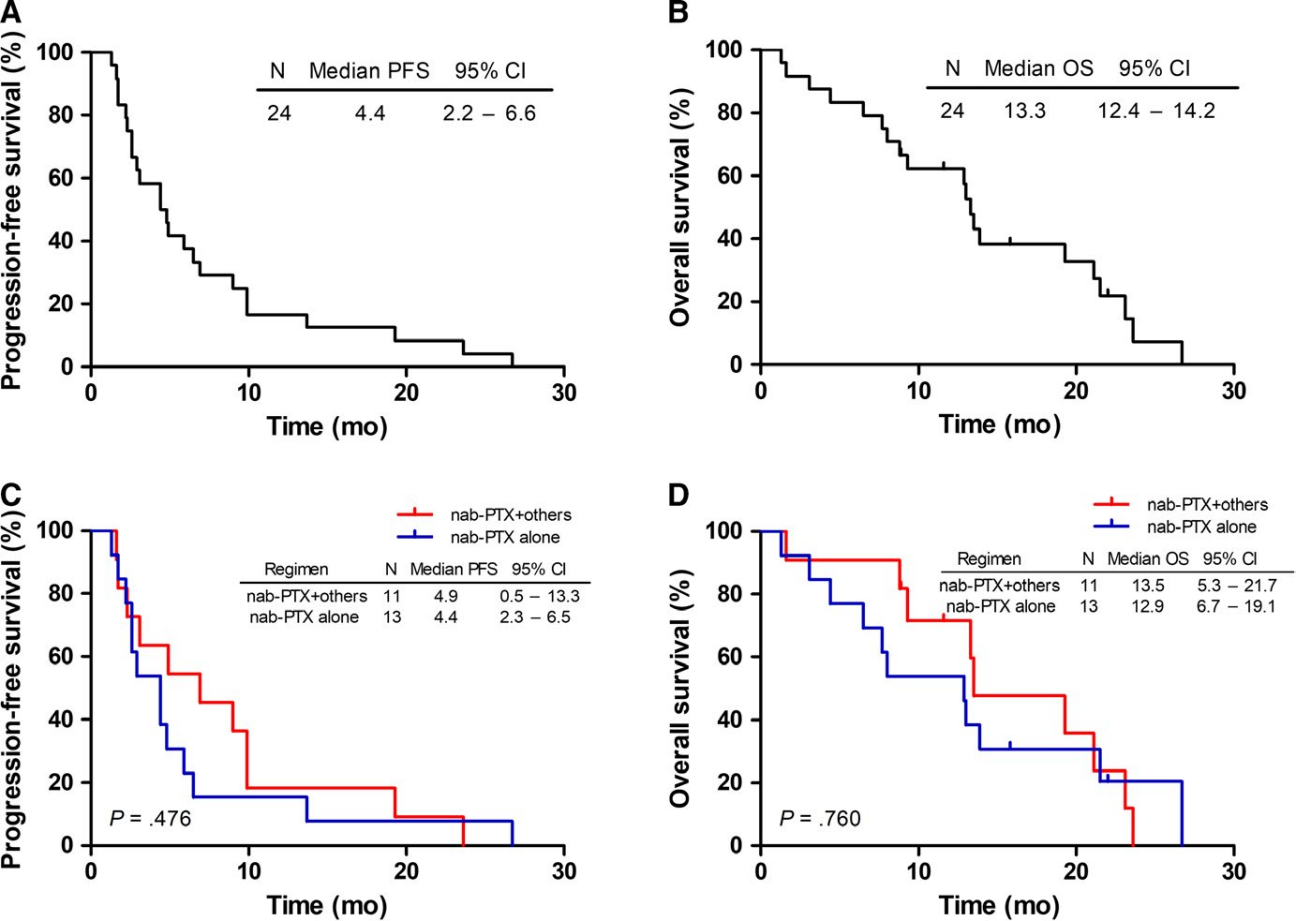
Kaplan‐Meier curves of progression‐free survival (PFS) and overall survival (OS) for patients who received nab‐PTX as second‐line or later treatment. A, The PFS curves for these subgroup patients. B, The OS curves for these subgroup patients. C, The PFS curves for patients who received nab‐PTX monotherapy or combination chemotherapy. D, The OS curves for patients who received nab‐PTX monotherapy or combination chemotherapy

Previous treatments were shown in Supplementary Table S1. The nab‐PTX‐based regimens were as first‐line chemotherapy in 28 (53.8%), second‐line in 17 (32.7%), third‐line in three (5.8%), and fourth‐line in four (7.7%) patients. As shown in Figure 2, for patients with nab‐PTX as first‐line treatment, the median PFS and OS were 6.7 months (95% CI: 5.9‐7.5 months) and 17.2 months (95% CI: 10.8‐23.6 months), respectively. In these patients, the median OS within combination chemotherapy subgroup was significantly longer than that in the monotherapy subgroup (20.3 vs 11.2 months, *P* = .013). Nevertheless, difference was not observed in the median PFS (7.1 vs 3.9 months, *P* = .249). In patients with nab‐PTX as second‐ or later‐line treatment, the median PFS and OS were 4.4 months (95% CI: 2.2‐6.6 months) and 13.3 months (95% CI: 12.4‐14.2 months), respectively; no difference was noticed between combination chemotherapy and monotherapy in survival (Figure 3).

### 3.3 Adverse events outcomes

The recorded AEs are listed in Table 4. For hematological AEs, grade 3/4 neutropenia and thrombocytopenia were observed in seven (13.5%) and two (3.8%) patients, respectively, and there were three (5.8%) patients recorded with grade 3/4 anemia. All hematological AEs were resolved by treatment. For nonhematologic toxicities, the most frequent AEs observed were peripheral sensory neuropathy (42.3%), followed by anorexia (40.4%) and nausea/vomiting (34.6%). Grade 3/4 nonhematologic toxicities mainly included peripheral sensory neuropathy (5.8%), nausea/vomiting (1.9%), and myalgia/arthralgia (1.9%). All nonhematological AEs were manageable. No treatment‐related deaths happened.

**Table 4:** Adverse events related to nanoparticle albumin‐bound paclitaxel

Adverse events	No. of patients (n = 52)					
	Grade				All grades	Grade 3/4
	1	2	3	4	n (%)	n (%)
Hematologic						
Anemia	9	6	1	2	18 (34.6)	3 (5.8)
Neutropenia	9	10	1	6	26 (50.0)	7 (13.5)
Thrombocytopenia	5	5	1	1	12 (23.1)	2 (3.8)
Nonhematologic						
Peripheral sensory neuropathy	12	7	3	0	22 (42.3)	3 (5.8)
Anorexia	15	6	0	0	21 (40.4)	0 (0)
Nausea/vomiting	13	4	1	0	18 (34.6)	1 (1.9)
Diarrhea	4	1	0	0	5 (9.6)	0 (0)
Constipation	5	1	0	0	6 (11.5)	0 (0)
Myalgia/arthralgia	9	4	1	0	14 (26.9)	1 (1.9)
Alopecia	14	1	0	0	15 (28.8)	0 (0)
Fatigue	9	4	0	0	13 (25.0)	0 (0)
Elevated ALT/AST	7	1	0	0	8 (15.4)	0 (0)
Mucositis	4	0	0	0	4 (7.7)	0 (0)
Skin rash	1	0	0	0	1 (1.9)	0 (0)

## 4 DISCUSSION

Our results revealed that when treated with nab‐PTX chemotherapy regimens, ORR and DCR in elderly advanced squamous NSCLC patients aged ≥70 were 34.6% and 80.8%, respectively. And, the median PFS and OS were 5.9 and 14.3 months. Additionally, patients who received combination chemotherapy regimens had significantly longer OS and a trend toward improved PFS compared to those who received nab‐PTX monotherapy, in both the total studied subjects or the subgroup with nab‐PTX as first‐line treatment. The incidence of toxicities with grade 3 or 4 was low and all AEs were manageable. These results suggested good tolerability and treatment response of nab‐PTX in elder advanced lung SCC patients.

The nab‐PTX is a solvent‐free paclitaxel, which can reach tumor environment in a higher local concentration and has fewer side effects. Many early studies and clinical trials have assessed the activity of nab‐PTX as first‐line treatment regimens in advanced NSCLC patients.^18^, ^24^ A previous trial in phase III demonstrated that, when compared to sb‐P/C, nab‐P/C had achieved a significantly fewer grade 3/4 AEs but higher ORR in treating advanced NSCLC patients as the first‐line treatment.^19^ And, stratified analysis showed that nab‐P/C led to a 1‐month increased median OS and a 68% improved response rate in SCC patients.^20^ Furthermore, Mudad et al compared gemcitabine plus cisplatin (G/C) to nab‐P/C in metastatic or advanced lung SCC and observed that the group of nab‐P/C had a significantly better median OS (12.8 vs 9.0 months, *P* = .03) and lower grade 3 or 4 toxicities than the G/C group.^25^ Based on these data, the nab‐PTX's safety and efficacy have been approved and accepted for the first‐line treatment in advanced squamous NSCLC. Our present analysis showed an even longer PFS and OS in elderly lung SCC patients with nab‐PTX as first‐line treatment.

The advanced squamous NSCLC's second‐line treatment, often aiming to alleviate symptoms and prolong the survival of patients, is considered more difficult. To date, various agents, such as docetaxel,^26^ nivolumab, and pembrolizumab, have been proved effective for recurrent NSCLC. For instance, as a second‐line therapy, single‐agent docetaxel yielded improved PFS (10.6 vs 6.7 weeks, *P* < .001) and OS (7.0 vs 4.6 months, *P* = .047) compared with best supportive care.^26^ Nivolumab and pembrolizumab, as anti‐programmed death 1 (anti‐PD‐1) immune checkpoint inhibitors, have been proved to increase PFS (3.5 and 3.7 months, respectively) and OS (9.2 and 12.0 months, respectively) in treating NSCLC patients as second‐line treatment.^27^, ^28^, ^29^ Furthermore, according to the analysis of CheckMate 017 study, compared with docetaxel, nivolumab has achieved a better OS (9.2 vs 6.0 months) and ORR (20% vs 9%, *P* = .0083) in recurrent squamous NSCLC patients.^27^ Some previous studies have also proved that nab‐PTX was efficacious as second‐line treatment for NSCLC patients.^30^, ^31^ Our present study displayed a median PFS of 4.4 months and a median OS of 13.3 months, which indicated that nab‐PTX might also have promising antitumor effect for elderly lung SCC patients who experienced previous treatment failure. Previous studies have reported that, combination chemotherapy, when compared with single‐agent chemotherapy, could not achieve survival benefit as second‐line treatment of advanced NSCLC^32^ and this was also observed in the present study in elderly squamous NSCLC patients.

Approximately 40% of all NSCLC patients are diagnosed in older adults ≥70, and the treatment of these elderly patients is an extremely challenging task^33^. Declined organ function and higher incidence of comorbidities may lead to intolerance to toxicity of chemotherapy and the limitation of therapeutic options, although the benefit has been proved by some studies in patients with good ECOG performance status.^34^, ^35^, ^36^ In the subset analysis of the phase III clinical trial conducted by Socinski et al, compared with enrolled older patients (≥70 years old) with sb‐P/C, the ORR of those with nab‐P/C was higher (34% vs 24%, *P* = .196). Additionally, the median PFS of nab‐P/C group was extended (8.0 vs 6.8 months, *P* = .134), and the median OS was significantly increased in the group of nab‐P/C (19.9 vs 10.4 months, *P* = .009).^21^ It is worth mentioning that in our study, the survival of patients with nab‐PTX‐based combination chemotherapy was very similar to the above results. And, we also observed a slightly higher ORR (36.7%) in combination chemotherapy regimens. All these results demonstrated that nab‐PTX plus other cytotoxic anticancer drugs, primarily platinum, could be more beneficial for elderly advanced squamous NSCLC patients with ages ≥70 years, and the toxicities of drugs could be well tolerated.

Our study has several limitations. Firstly, the number of patients was limited, which may lead to insufficient statistical power especially in the subgroup analysis. Secondly, in this retrospective study, a variety of factors could affect the choice of treatment by the physicians, including the physical condition, severity of disease, and economic condition, which may affect the evaluation of the effectiveness of nab‐PTX treatment in subgroups. Finally, due to the retrospective nature, data collection of AEs in patients may not be sufficient. Further randomized controlled studies, preferably in large‐scale studies, are needed to confirm the role of nab‐PTX in treating advanced lung SCC patients aged ≥70 years.

In summary, nab‐PTX appeared to be with high treatment response in elderly advanced squamous NSCLC patients ≥70 years old, in both first‐line and second‐ or later‐line chemotherapy. Meanwhile, given the improved ORR, PFS, and OS vs nab‐PTX monotherapy, nab‐PTX‐based combination chemotherapy regimens, especially nab‐PTX plus platinum, are recommended for older population with good baseline performance status.

## CONFLICT OF INTEREST

None declared.

## Data Availability

Data supporting this study are available upon request from the corresponding author. Due to privacy or ethical restrictions, these data are not publicly available.
