# Supplementary material for: Nanoparticle albumin‐bound paclitaxel in elder patients with advanced squamous non‐small‐cell lung cancer: A retrospective study
Source: Cancer Med. 2019 Dec 26;9(4):1365–73. doi: 10.1002/cam4.2791 (PMC7013054; doi:10.1002/cam4.2791)
Supplement: Supplementary file 1 [file CAM4-9-1365-s001.pdf]

Supplementary Table S1. Previous chemotherapy regimens for the patients age ≥70 years with squamous NSCLC included in the present study.

Case	First	Second	Third	Fourth
1	nab-PTX			
2	NVB+DDP	GEM+DTX	nab-PTX	
3	nab-PTX			
4	GEM	nab-PTX+DDP		
5	GEM	PTX	S-1	nab-PTX
6	nab-PTX			
7	nab-PTX			
8	nab-PTX			
9	GEM+DDP	nab-PTX		
10	nab-PTX+NDP			
11	nab-PTX+CBP			
12	nab-PTX+GEM			
13	VP-16+DDP	nab-PTX		
14	nab-PTX			
15	nab-PTX			
16	GEM	nab-PTX		
17	nab-PTX+CBP			
18	GEM+NDP	nab-PTX+DDP		
19	PEM	nab-PTX		
20	nab-PTX+DDP			
21	GEM	nab-PTX		
22	nab-PTX+DDP			
23	GEM+DDP	nab-PTX		
24	DTX+CBP	GEM+S-1	GEM+DDP	nab-PTX
25	GEM+DDP	nab-PTX+CBP		
26	GEM+CBP	nab-PTX		
27	GEM+DDP	nab-PTX		
28	nab-PTX+DDP			
29	GEM+NDP	nab-PTX		
30	DTX+CBP	nab-PTX+CBP		
31	nab-PTX+NDP			
32	nab-PTX+DDP			
33	nab-PTX+DDP			
34	nab-PTX			
35	nab-PTX+CBP			
36	nab-PTX+NDP			
37	GEM+NDP	nab-PTX		
38	nab-PTX+DDP			
39	nab-PTX+DDP			
40	nab-PTX+CBP			
41	PTX	nab-PTX		
42	nab-PTX+CBP			
43	GEM+DDP	PTX	DTX+DDP	nab-PTX+DDP
44	nab-PTX+NDP			
45	nab-PTX			
46	nab-PTX+DDP			
47	DTX+DDP	GEM+CBP	nab-PTX+DDP	
48	GEM+DDP	nab-PTX+CBP		
49	GEM+DDP	DTX+NDP	NVB+LBP	nab-PTX+CBP
50	GEM+DDP	nab-PTX+NDP		
51	GEM+CBP	DTX+NDP	nab-PTX+NVB	
52	nab-PTX+DDP			

PEM=pemetrexed, DTX=docetaxel, NVB=vinorelbine, GEM=gemcitabine, PTX=paclitaxel, nab-PTX=nanoparticle albumin-bound PTX, VP-16=etoposide, DDP=cisplatin, CBP=carboplatin, NDP=nedaplatin, LBP=lobaplatin.
